# Different Dimensional Copper-Based Metal–Organic Frameworks with Enzyme-Mimetic Activity for Antibacterial Therapy

**DOI:** 10.3390/ijms24043173

**Published:** 2023-02-06

**Authors:** Chuyan Lin, Xiangjian Guo, Fayin Mo, Duanping Sun

**Affiliations:** 1 Guangdong Provincial Key Laboratory of Pharmaceutical Bioactive Substances, Center for Drug Research and Development, Guangdong Pharmaceutical University, Guangzhou 510006, China; 2 Key Specialty of Clinical Pharmacy, The First Affiliated Hospital of Guangdong Pharmaceutical University, Guangzhou 510699, China; 3 The First Affiliated Hospital of Sun Yat-sen University, Guangzhou 510080, China

**Keywords:** metal–organic frameworks, POD-like activity, antibacterial therapy, wound healing

## Abstract

Fighting against bacterial infection and accelerating wound healing remain important and challenging in infected wound care. Metal–organic frameworks (MOFs) have received much attention for their optimized and enhanced catalytic performance in different dimensions of these challenges. The size and morphology of nanomaterials are important in their physiochemical properties and thereby their biological functions. Enzyme-mimicking catalysts, based on MOFs of different dimensions, display varying degrees of peroxidase (POD)-like activity toward hydrogen peroxide (H_2_O_2_) decomposition into toxic hydroxyl radicals (•OH) for bacterial inhibition and accelerating wound healing. In this study, we investigated the two most studied representatives of copper-based MOFs (Cu-MOFs), three-dimensional (3D) HKUST-1 and two-dimensional (2D) Cu-TCPP, for antibacterial therapy. HKUST-1, with a uniform and octahedral 3D structure, showed higher POD-like activity, resulting in H_2_O_2_ decomposition for •OH generation rather than Cu-TCPP. Because of the efficient generation of toxic •OH, both Gram-negative Escherichia coli and Gram-positive methicillin-resistant *Staphylococcus aureus* could be eliminated under a lower concentration of H_2_O_2_. Animal experiments indicated that the as-prepared HKUST-1 effectively accelerated wound healing with good biocompatibility. These results reveal the multivariate dimensions of Cu-MOFs with high POD-like activity, providing good potential for further stimulation of specific bacterial binding therapies in the future.

## 1. Introduction

Metal–organic frameworks (MOFs) are crystalline porous materials constructed by the coordination of metal ions or clusters with polytypic organic ligands [1,2]. They possess many promising features, such as tunable structures, active sites, rapid electron transmission, and high surface area [3,4]. Owing to their excellent physical and chemical properties, MOFs have been extensively used in electrochemical applications, gas storage, and biomedical fields, such as wound healing, enhanced cancer therapy, imaging, and antibacterial agents [5,6,7,8]. As nanozymes, MOFs have been widely explored to achieve better antibacterial efficiency due to their peroxidase (POD)-like activity [9]. They can catalyze hydrogen peroxide (H_2_O_2_) into hydroxyl radicals (•OH), which possess higher antibacterial activity and minimize the toxicity of higher concentrations of H_2_O_2_ [10]. Copper-based MOFs (Cu-MOFs) stand out among numerous MOFs due to their low cost, exceptional stability, environmental friendliness and nontoxicity [11]. On the one hand, the intrinsic antibacterial activity of Cu^2+^ renders Cu-MOFs into an antibacterial platform, either alone or in combination with other functional ligands and antibacterial composites. Shams et al. found that pure HKUST-1 displayed a notable antibacterial effect against Escherichia coli (*E. coli*) and *S. aureus* by significantly disrupting the cell membrane, discharging cell constituents, and inhibiting DNA synthesis [12]. On the other hand, the POD-like catalytic effect of Cu-MOFs could be harnessed to construct antibacterial composites. POD-like activity can kill bacteria by converting H_2_O_2_ into toxic •OH at low concentrations, producing bactericidal reactive oxygen species (ROS) through the Fenton-type reaction to damage the cell membrane directly [13,14]. Significantly, MOFs with more powerful POD-like activity were able to destroy the exposed bacteria, avoiding the decolonization of bacteria [10,15]. According to the differences in morphology, Cu-MOFs can be categorized into 1-dimensional (1D), two-dimensional (2D), and three-dimensional (3D) MOFs [16,17,18]. The size and morphology of nanomaterials are important in their physiochemical properties and thereby their biological functions [19]. For example, the uniform dimensions of MOFs are essential for their applications in drug delivery and bioassays, as their size distribution may affect the biodistribution and linear quantitative analysis of drugs [20,21]. The high surface energy of MOFs leads to their propensity to aggregate into larger and irregular sizes [19]. Cu-MOFs have excellent physicochemical properties among MOFs and excellent catalytic activity, properties which have recently attracted much attention in various biomedical applications [15,22]. The catalytic activity and substrate selectivity of the nanozyme can be reasonably tuned through prestructural design and modulation of the preparation process [15]. The catalytic activity of the nanozyme is dependent on multiple factors, such as the category, size, surface, and crystal structure of the nanomaterial; thus, the measurement and evaluation of catalytic activity are complicated. However, the comparative enzymatic catalytic activity of 2D and 3D MOFs in different dimensions for biomedical applications has rarely been reported and thus is not preferred.

Bacterial infections have been one of the most concerning issues in the clinical medicine field and have become an urgent global hazard to public health, bringing a growing challenge to healthcare systems [23,24]. Bacterial infections can cause many serious and related diseases, such as delayed wound healing, bacteremia, and even death [25,26]. In particular, acute and chronic wounds due to all kinds of injuries are accompanied by bacterial infections [24]. The two common bacteria in wound sites are the Gram-positive bacterium methicillin-resistant *Staphylococcus aureus* (MRSA) and Gram-negative bacterium Pseudomonas aeruginosa [27,28]. Currently, antibiotics are the traditional method of infection therapy. However, the abuse and prolonged use of antibiotics can result in drug resistance in bacteria and even super bacteria, threatening human health and causing a huge medical cost burden [29,30]. According to a recent study, more than 700,000 people die worldwide annually from antibiotic-resistant infections, and this number is expected to rise to 10 million a year by 2050 [31]. Thus, the development of antibacterial therapy against complex infections at the wound site is urgently essential in biomedical applications [32].

**Scheme 1:**
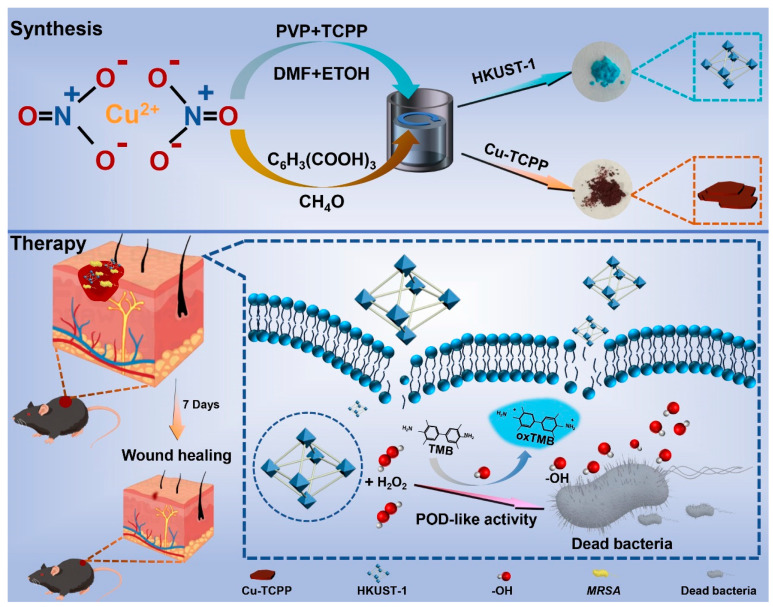
Schematic illustration of the preparation of two different dimensional Cu-MOFs with POD-like activity for antibacterial and wound healing.

HKUST-1 and Cu-TCPP MOFs are the most widely explored 3D and 2D Cu-MOFs, respectively [18,33,34]. Here, we report the POD-like activities of Cu-MOFs, with different dimensions (Cu-TCPP/HKUST-1) for potential antimicrobial agents with low biotoxicity and high antimicrobial activity. The POD-like activity of 2D/3D Cu-MOFs in antimicrobial therapy was compared. (Scheme 1) Briefly, we developed a straightforward synthetic strategy to prepare Cu-TCPP and HKUST-1 and demonstrated their successful synthesis by characterization techniques. HKUST-1 showed better POD-like activity due to its uniform and octahedral structure for accelerating electron transportation and ROS generation. HKUST-1 could capture Gram-negative *E. coli* and Gram-positive MRSA bacteria through ROS destruction and kill some bacteria by the disintegration of H_2_O_2_ into toxic •OH. Moreover, HKUST-1 exhibits excellent biocompatibility. They significantly accelerate the healing of wounds infected with MRSA bacteria. We compared the bactericidal effect based on the catalytic performances of 2D and 3D Cu-MOFs and highlighted the promising potential of Cu-MOFs for POD-like antibacterial wound healing treatment. This work provides a strategy to develop multi-dimension MOFs that target bacteria, which will further inspire specific bacterial-binding therapy in the future.

## 2. Results and Discussion

### 2.1. Characterization of Cu-MOFs

**Figure 1:**
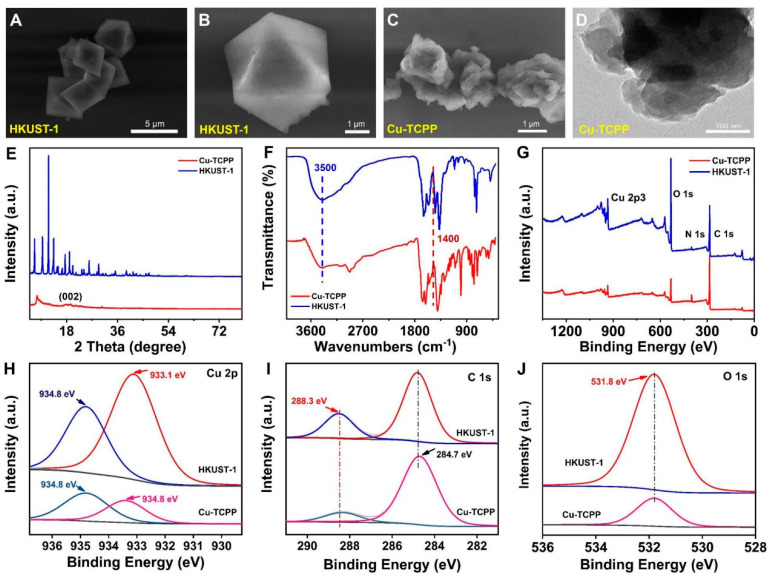
(**A**,**B**) SEM image of HKUST-1. (**C**) SEM image of Cu-TCPP. (**D**) TEM image of Cu–TCPP. (**E**) XRD patterns and (**F**) FT-IR spectra of HKUST-1 and Cu-TCPP. (**G**) XPS survey spectra for HKUST-1 and Cu-TCPP. High-resolution XPS spectra of (**H**) Cu 2p, (**I**) C 1s and (**J**) O 1s for HKUST-1 and Cu-TCPP.

To study the surface morphology of Cu-MOFs, the obtained materials were characterized by scanning electron microscopy (SEM) and transmission electron microscopy (TEM). The morphology of Cu-MOFs is displayed in Figure 1. As shown in Figure 1A,B, the prepared HKUST-1 presented a uniform and 3D octahedral shape structure with an average size of 1–3 μm. A magnified SEM image reveals that the octahedral-shaped HKUST-1 possesses rough surfaces with porous structures. It reveals the possibility of providing a high surface area for substance transmission, catalysis and antibacterial applications. Similarly, the prepared Cu-TCPP was characterized by SEM and TEM, and the images are shown in Figure 1C and Figure 1D, respectively. The obtained Cu-TCPP proved a sheet-like and layer-by-layer ultrathin 2D structure with a wrinkled film surface and folds, suggesting 2D Cu-TCPP nanosheets with a large surface area. The two kinds of Cu-MOFs were in accordance with the previous literature. Supplementary Figure S1A,B show the powders of the prepared Cu-MOFs.

The crystalline structures of the as-prepared Cu-MOFs were investigated by X-ray diffraction (XRD). The intact diffraction characteristic peak profiles of Cu-TCPP and HKUST-1 from 2θ = 5–80°. Cu-TCPP exhibited a distinguishing peak at 2θ = 20°, which can be indexed to the (002) crystal plane (Figure 1E). The XRD pattern of HKUST-1 was mainly composed of distinguishing peaks in the range of 2θ = 5°–20°, indicating the crystalline characteristics of the synthesized HKUST-1 samples. It revealed microporous coordination with the cubic crystalline structure and high crystallinity. The diffraction pattern was consistent with that reported in the literature, suggesting the successful synthesis of Cu-TCPP and HKUST-1. Further information about the chemical composition in terms of functional groups on Cu-MOFs was provided by Fourier transform infrared (FT-IR) spectra presented in Figure 1F. The bands in the range of 700 to 1700 cm^−1^ were assigned to BTC, and the characteristic peak at approximately 500 cm^−1^ was contributed by Cu-O stretching vibrations. The spectra of Cu-TCPP and HKUST-1 presented two strong peaks at approximately 1400 and 1620 cm^−1^. The most significant characteristic peak at 3500 cm^−1^ was contributed by HKUST-1. The FT-IR spectrum of HKUST-1 demonstrated an almost isobidentate behavior of the COO moiety since bands at 1645, 1620, 1570, 1550, 1445, and 1375 cm^−1^ are characteristic bands of this coordination mode. The bands at 1445 and 1645 cm^−1^ indicated –O–C–O– bonding as well as those at 1375 and 1550 cm^−1^ of C=C stretching, demonstrating the incorporation of BTC in the MOF [17]. The latter is because aniso-bidentate dicopper (II) carboxylate is a type of monomeric cluster present in the framework. X-ray photoelectron spectroscopy (XPS) was used to analyze the surface composition, chemical composition, and states of Cu-MOFs. The surface characteristics of the obtained materials were investigated by XPS. Figure 1G–J demonstrates a full survey of Cu-TCPP and HKUST-1 composed of Cu 2p3, O 1s, C 1s, and N 1s (Figure S2). In the Cu 2p3 region, the patterns of HKUST-1 and Cu-TCPP show characteristic peaks at approximately 900 eV (Figure 1H). These results suggested that two kinds of Cu-MOFs were synthesized successfully.

### 2.2. POD-like Catalytic Activity of Cu-MOFs

**Figure 2:**
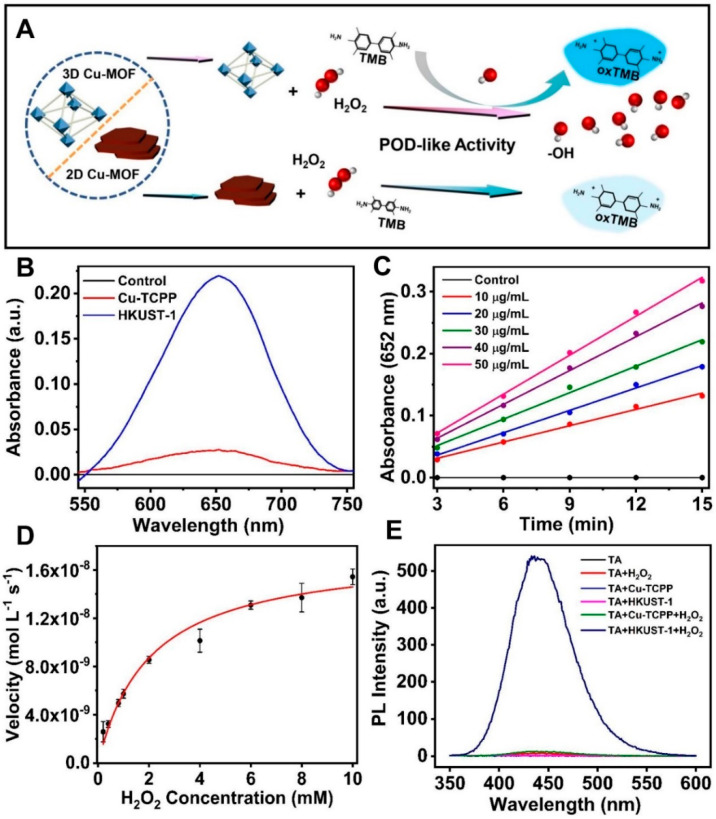
(**A**) Schematic diagram of the mechanism for POD-like activity in two different dimensions of MOF. (**B**) The UV–vis absorbance spectra of TMB in different reaction systems: H_2_O_2_ + TMB, Cu-TCPP + H_2_O_2_ + TMB, and HKUST-1 + H_2_O_2_ + TMB. (**C**) Kinetic curves of prepared Cu-MOFs artificial enzyme with varied concentrations. (**D**) Steady-state kinetic assays of HKUST-1 for H_2_O_2_. (**E**) Fluorescence spectra of different reaction systems: TA, TA + H_2_O_2_, TA + Cu-TCPP, TA + HKUST-1, TA + H_2_O_2_ + Cu-TCPP and TA + H_2_O_2_ + HKUST-1.

In the first suite of experiments, we used H_2_O_2_ and 3,3′,5,5′-tetramethylbenzidine (TMB) to evaluate the catalytic ability of POD-like enzymes as substrates to challenge the catalytic ability of the prepared Cu-MOFs. The decomposition of H_2_O_2_ can be accelerated by catalysts to generate more oxygen, leading to gas bubble formation. Figure S3 displays a comparison of the gas bubble formation with the assistance of Cu-TCCP and HKUST-1. Both Cu-TCPP and HKUST-1 presented catalase-like activity with gas bubble formation. More gas bubbles were produced in tubes containing H_2_O_2_ + HKUST-1 (Figure S3-III), suggesting a higher catalase-like activity than that of Cu-TCPP. TMB is a typical chromogenic substrate serving as an indicator to determine POD-like activity. H_2_O_2_ could be degraded to generate •OH with the assistance of POD, which activated the cascade production of blue oxidization TMB (oxTMB) with a color change of the solutions. The POD-like catalytic activity of Cu-MOFs was studied by the colorimetric oxidation reaction system of TMB in the absence or presence of H_2_O_2_ (Figure 2A). Negligible color changes could be seen from the tubes in the absence of Cu-MOFs (Figure S4-II). In contrast, obvious changes from colorless to blue can be observed from the tubes containing Cu-MOFs, TMB, and H_2_O_2_ (Figure S4-V,VI), indicating the oxidation process with the production of oxTMB. All the results suggested that HKUST-1 with better POD-like catalytic activity can be used as a catalyst for the acceleration of TMB oxidization. Furthermore, UV–visible spectroscopy was employed to quantify TMB oxidation by H_2_O_2_ with the assistance of Cu-MOFs. The maximum characteristic absorption peak of oxTMB can be observed at 652 nm. Significantly, the TMB + H_2_O_2_ + HKUST-1 reaction system presented a higher absorption peak in comparison with TMB + H_2_O_2_ + Cu-TCPP, revealing the better POD-like catalytic activity of HKUST-1 than Cu-TCPP (Figure 2B). The excellent POD-like catalytic activity could be attributed to the unique 3D structure of HKUST-1 to expose more tunnel and catalytic active sites for substance exchange, which considerably enhanced the catalytic performance. We systematically explored the effect of the concentrations of catalyst and reaction time. The characteristic absorption peak value at 652 nm increased linearly with increasing HKUST-1 concentration and reaction time (Figure 2C). The TMB oxidation reaction in the presence of H_2_O_2_ with the assistance of HKUST-1 occurs in a catalyst concentration- and time-dependent manner (Figure S5). HKUST-1 was selected as an ideal POD-like catalyst in the following experiment based on the above results.

To analyze the POD-like catalytic activity as well as the catalytic mechanism, the kinetic parameters of HKUST-1 were further quantitatively studied using enzyme kinetics theory. Under the optimal reactive conditions, the steady-state kinetic properties of HKUST-1 were studied by changing the concentration of substrate (TMB and H_2_O_2_) with different reaction times. In this typical enzyme reaction kinetics, the concentration of HKUST-1 was fixed. The characteristic absorption peak value at 652 nm was recorded by fixing the concentration of TMB as well as changing the concentration of H_2_O_2_ in a specified range and vice versa with H_2_O_2_. The absorbance at 652 nm was enhanced with increasing concentrations of H_2_O_2_ and TMB. Figure S6 shows that the reaction rate of HKUST-1 increased obviously with increasing TMB when the concentration of H_2_O_2_ was fixed. With further growth of the concentration of TMB, the reaction rate increased slowly and flattens out gradually. Similarly, when the concentration of TMB was fixed, the reaction rate of HKUST-1 POD gradually increased with increasing H_2_O_2._ However, the reaction rate slowed down and flattened out gradually with a further extension of the concentration of H_2_O_2_ (Figure 2D). The results showed that the TMB oxidation reaction catalyzed by HKUST-1 followed the typical Michaelis–Menten behavior toward H_2_O_2_ and TMB. Table 1 lists a comparison of the $K_{m}$ and $V_{max}$ values of as-prepared HKUST-1 and several catalysts in previous reports for substrates TMB and H_2_O_2_. The $K_{m}$ value of HKUST-1 was determined to be 2.036 mM for the H_2_O_2_ substrate and 0.545 mM for the TMB substrate, indicating that HKUST-1 possessed a good affinity for H_2_O_2_ and TMB. Compared with HKUST-1 and other reported catalysts, the $K_{m}$ value of HKUST-1 with H_2_O_2_ as a substrate was lower than that of ZIF-67 (3.52 mM), antibody@Cu-MOFs (7.37 mM) and Cu-MOFs (CuCl_2_) (6.41 mM), demonstrating a higher POD-like activity toward the TMB-H_2_O_2_ reaction system. This could benefit from the large surface areas, rough surface, pore sizes and tunnel in HKUST-1, accelerating the substance exchange. The HKUST-1 with unique 3D structure displays higher POD-like activity.

### 2.3. Detection of •OH

To further investigate the catalytic mechanism, a fluorescence experiment was carried out to analyze the POD-like activity of Cu-MOFs. Terephthalic acid (TA) was employed as a fluorescent indicator to further detect the generation of •OH, decomposed from H_2_O_2_ with the catalysis of Cu-MOFs. The detection principle says that •OH can be captured by the nonfluorescent compound TA to generate a highly fluorescent product, 2-hydroxyterephthalic acid (TAOH), with a unique characteristic fluorescence peak at 435 nm under excitation at 315 nm (Figure 2E). The fluorescence signal can be recorded by the spectrofluorometer to confirm the generation of •OH. Figure 2E shows the fluorescence spectra of Cu-MOFs, TA, H_2_O_2_, and their mixture in comparison with the control groups after incubation for 12 h in the dark, demonstrating the generation of •OH. Compared with the TA + H_2_O_2_ + HKUST-1 group, the fluorescence intensity of the TA + H_2_O_2_ + Cu-TCPP group displayed an unsatisfying result, with a negligible fluorescent signal as well as the result of the control group. The emission intensity of TA increased obviously with the addition of HKUST-1. The fluorescence intensity of the TA + H_2_O_2_ + HKUST-1 group reached approximately 550, which was 50 times higher than that of the TA + H_2_O_2_ + Cu-TCPP group. The results revealed that HKUST-1, with excellent POD-like activity due to its unique 3D structure, can accelerate the decomposition of H_2_O_2_ for •OH generation.

### 2.4. Antibacterial Assay In Vitro

**Figure 3:**
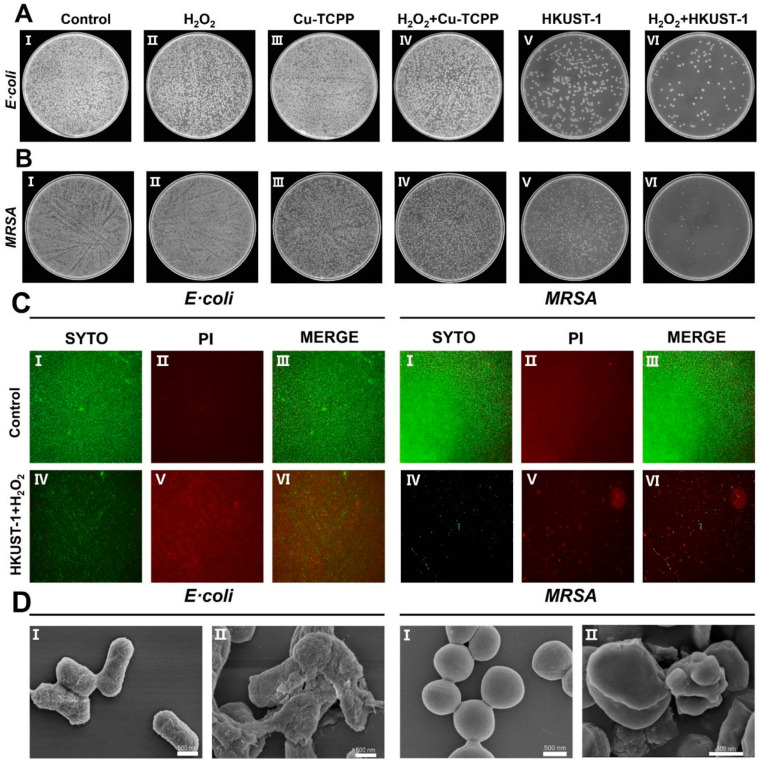
Photographs of the colonies against *E. coli* (**A**) and *MRSA* (**B**) with different treatments, (I) PBS (II) H_2_O_2_ (III) Cu-TCPP (IV) Cu-TCPP + H_2_O_2_ (V) HKUST-1 (VI) HKUST-1 + H_2_O_2_. (**C**) Fluorescence images of *E. coli* and *MRSA* that had undergone various treatments (I), (II) and (III) PBS and (IV), (V) and (VI) HKUST-1 + H_2_O_2_. (**D**) SEM images of *E. coli* and *MRSA* by various treatments: (I) PBS, (II) HKUST-1 + H_2_O_2_. Scale bar = 500 nm.

High concentrations of H_2_O_2_ have been widely used in sterilization and bacterial infectious therapy. However, it causes serious oxidative stress in tissues, which may cause unnecessary damage in tissues. In light of the impressive POD-like activity of HKUST-1 that could convert H_2_O_2_ into •OH, toxic •OH is much more reactive and can cause more serious oxidative damage to bacteria. We analyzed the antibacterial effects of Cu-MOFs in two dimensions against Gram-negative strains of *E. coli* and Gram-positive strains of MRSA. The antibacterial performance of the Cu-MOFs in two dimensions was evaluated by the plate counting method. Six different groups were set for exploring the antibacterial performance against both Gram-negative and Gram-positive pathogens, including (I) PBS, (II) H_2_O_2_, (III) Cu-TCPP, (IV) HKUST-1, (V) Cu-TCPP + H_2_O_2_ and (VI) HKUST-1 + H_2_O_2_ groups. Two kinds of bacteria were treated with different treatments before inoculation onto LB culture plates, and the PBS group was set as a parallel control. According to Figure 3A,B, HKUST-1 illustrated little antibacterial ability against *E. coli* and MRSA in the absence of H_2_O_2_, demonstrating that HKUST-1 has weak antibacterial activity. This could be attributed to the copper ions released from HKUST-1. As expected, both kinds of bacteria incubated with HKUST-1 + H_2_O_2_ treatments presented a dramatically decreasing trend, demonstrating the outstanding antibacterial activity contributed by the effect of HKUST-1 + H_2_O_2_. The antibacterial performance of low concentrations of H_2_O_2_ was greatly enhanced with the addition of HKUST-1, avoiding damage during disinfection with high concentrations of H_2_O_2_. In comparison, no obvious change in the number of bacterial colonies was observed after treatment with PBS, H_2_O_2_, Cu-TCPP and Cu-TCPP + H_2_O_2_, which was consistent with the results of the POD-like activity assay. These results revealed a weak antibacterial activity and poor growth inhibition of Cu-TCPP compared to HKUST-1. Benefiting from the striking POD-like activity of HKUST-1 with a unique 3D structure, H_2_O_2_ could generate reactive and toxic •OH to combat bacteria. These findings could be ascribed to the intrinsic bactericidal activity of •OH as a result of the effective catalysis from HKUST-1.

After displaying the antibacterial performances of Cu-MOFs with the plate counting method, we further analyzed the potential antibacterial mechanism derived from HKUST-1. We employed live/dead viability analysis using confocal fluorescence microscopy. SYTO/propidium iodide (PI) staining was used to display the live/dead assays. The SYTO dye penetrated and stained both intact membranes and broken membranes, causing fluorescent green emission, while the PI dye penetrated only the damaged cell membranes, resulting in fluorescent red emission. The bright green fluorescence obtained from the SYTO stain indicated the negligible antibacterial effect of the PBS treatment. However, upon cotreatment with HKUST-1 and H_2_O_2_, the sharply predominant red fluorescence intensity of PI could be recorded, indicating damaged cell membranes and bacterial apoptosis (Figure 3C). This result adequately validated the excellent antibacterial performance due to the generation of highly toxic •OH benefiting from HKUST-1 with POD-like activity. The antibacterial effect shown in these experiments illustrated the potential application of the proposed combined treatment for combating bacterial infections, which further confirmed that H_2_O_2_ at very low concentrations can still yield effective antibacterial efficiency with the assistance of HKUST-1.

To further confirm the above results, SEM characterization was employed to visualize the morphological changes of bacteria with different treatments. As shown in Figure 3D, both Gram-negative strains of *E. coli* and Gram-positive strains of MRSA treated with PBS were typically rod-shaped and round-shaped, respectively. The smooth, uniform and intact morphologies of cells with few disruptions could be observed, indicating the negligible bactericidal function of PBS for combating bacteria. Compared to the control group, bacteria treated with HKUST-1 + H_2_O_2_ exhibited an obvious morphological change. The Gram-negative strains of *E. coli* displayed collapsed, split, and merged membranes, resulting in wrinkled and shrunken morphology after exposure to HKUST-1 + H_2_O_2_ treatment. Similar morphological changes were also observed for MRSA, which were consistent with the results of the plate counting method and the live/dead assays. Therefore, SEM characterization of the bacteria fully confirmed the outstanding bactericidal performance of HKUST-1, leading to the severe structural deformation of the bacteria. These results suggested that HKUST-1 disrupted the bacterial membrane and induced bacterial apoptosis. HKUST-1 with excellent POD-like activity to generate •OH, revealing its highly efficient bactericidal performance under a low concentration of H_2_O_2_.

### 2.5. Evaluation of Antibacterial Activity In Vivo

**Figure 4:**
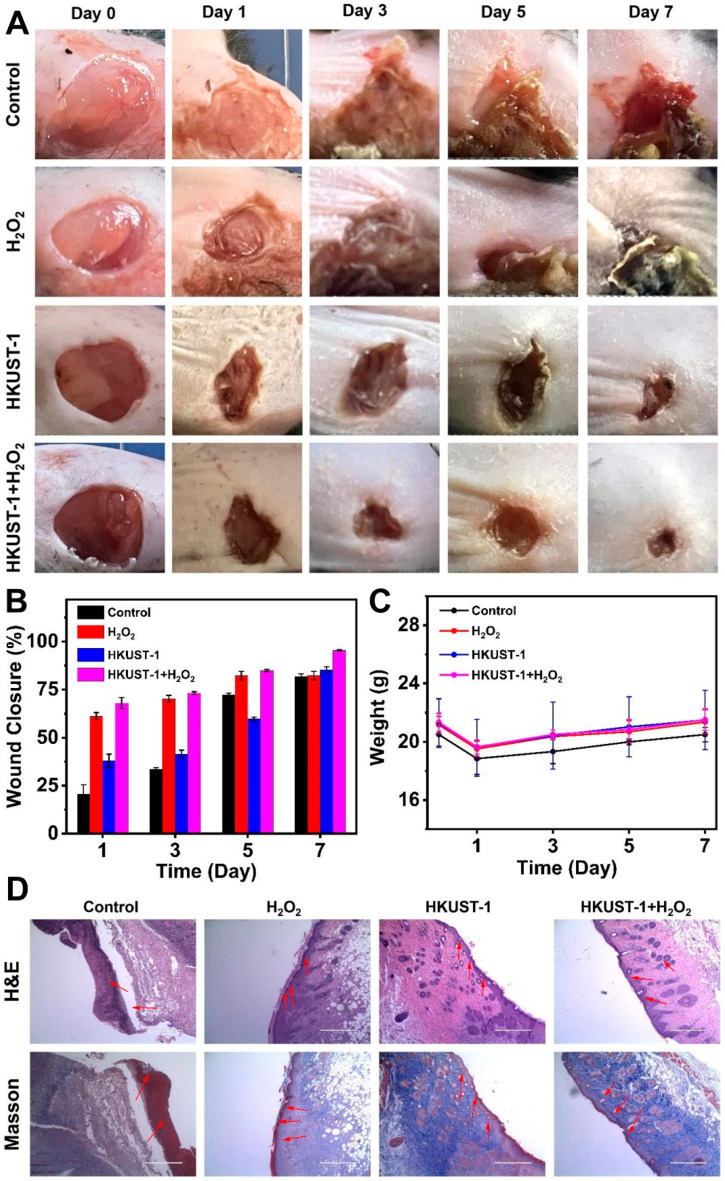
(**A**) Digital photos of MRSA-infected wounds on mice with therapy at different times. (**B**) Relative wound closure size of mice after different therapies. (**C**) Body weight of mice treated with different therapies for 0–7 days. (**D**) Corresponding H&E and Masson staining of skin tissue from mice with different therapies at day 7. The red arrow indicates the edge of the skin wound. Scale bar = 400 μm.

Bacterial infections can result in inflammatory reactions and postpone wound healing. Inspired by the remarkable antibacterial performance of HKUST-1 in vitro, we further investigated the bactericidal efficacy of HKUST-1 in vivo to determine its potential for accelerating infectious wound healing and clinical applications. An 8 mm-diameter circular full-thickness cutaneous wound model with *MRSA* infection was constructed on the backs of mice in this experiment section. First, 50 μL 1 × 10^7^ CFU mL^−1^ of MRSA suspension was dropped on the wound site to fabricate the infectious wound in each mouse, and the wounds were incubated with MRSA for 24 h. Subsequently, twenty C57BL/6 mice with an infectious wound on their backs were randomly divided into four groups: (I) PBS + 3% carboxymethyl cellulose sodium (CMC-Na) Hydrogels, (II) H_2_O_2_ + 3% CMC-Na Hydrogels, (III) HKUST-1 + 3% CMC-Na Hydrogels and (IV) HKUST-1 + H_2_O_2_ + 3% CMC-Na Hydrogels (Figure S7). Each group of wounds received different treatments for 7 consecutive days, and the hydrogels were changed at daily intervals after irrigation with physiological saline solution. Photographs of the wounds were taken by camera on each alternate day to demonstrate the therapeutic effects visually. As shown in Figure 4A, all wounds had obvious inflammatory reactions (red and swollen skin around the wound) after surgery, suggesting successful model construction. With the continuous process of the treatments, all groups with different treatments displayed decreased surface area of the wounds. However, the wounds treated with PBS + 3% CMC-Na hydrogels exhibited a relatively slow wound healing process. In particular, ulcers could be observed around the wound site on day 5 and day 7, indicating severe infections. A similar tendency could be seen in the treatment of H_2_O_2_ + 3% CMC-Na hydrogels. This result could be attributed to the negligible antibacterial effect of PBS and the low concentration of H_2_O_2_. Notably, HKUST-1 + H_2_O_2_ + 3% CMC-Na hydrogels significantly accelerated the wound closure process. The wounds almost completely healed after 7 days of treatment with scab formation. On the seventh day, the wound treated with HKUST-1 + H_2_O_2_ + 3% CMC-Na hydrogels displayed the best wound contraction compared with the other groups. In addition, the relative wound closure of mice with different treatments was calculated to visually assess the therapeutic effects. Figure 4B demonstrates the relative wound closure of each group in the whole therapeutic process. Importantly, the wound treated with HKUST-1 + H_2_O_2_ + 3% CMC-Na hydrogels illustrated the highest relative wound closure at each observation period, and the relative wound closure was 90% at day 7. Furthermore, the toxicity of HKUST-1 was studied by the weight changes in mice during the therapeutic process. Notably, no obvious weight change could be observed in each group of mice, indicating the nontoxicity of the as-prepared HKUST-1 (Figure 4C).

Histological analysis of the wound tissues was conducted to study the acceleration of the wound-healing efficiency of HKUST-1 (Figure 4D). Hematoxylin and eosin staining (H&E) and Masson staining were used to assess skin tissue repair, collagen formation, epidermis architecture and inflammation levels after different treatments. The wound tissues of each group were collected from the sacrificed mice after 7 days of treatment. As shown in Figure 4D, the H&E staining exhibited intact epidermis architecture and few inflammatory cells on the wound, with normal skin thickness in the HKUST-1 + H_2_O_2_ + 3% CMC-Na hydrogel group. Similar results can be observed in Masson staining, and uniform collagen fiber deposition and generation of the epithelial layer were observed in the wound treated by HKUST-1 with the assistance of a low concentration of H_2_O_2_, revealing commendable antibacterial effects and promotion in the healing process. In contrast, a larger number of inflammatory cells were recruited at the site of the wound in the PBS +3% CMC-Na hydrogel group than in the other groups. This may have impaired the tissue in wound sites with fibrotic repair, oxidation and inflammatory cells, suggesting severe infection. Based on the above results, we can conclude that the HKUST-1 + H_2_O_2_ + 3% CMC-Na hydrogel group showed an excellent wound healing effect in vivo against MRSA infection due to serious oxidative damage, induced by highly toxic •OH, benefiting from HKUST-1 with higher POD-like activity.

### 2.6. Biosafety Assay In Vivo

**Figure 5:**
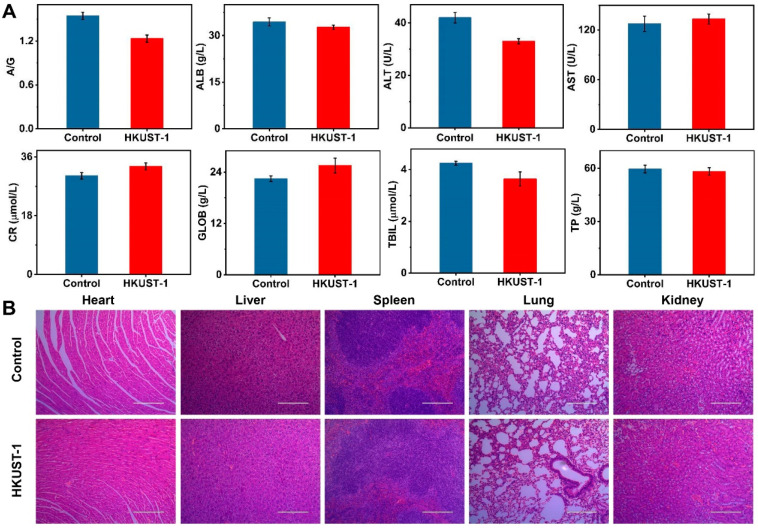
(**A**) Levels of different blood biochemical indexes (A/G, ALB, ALT, AST, CR, GLOB, TBIL, and TP) after intravenous injection with PBS solution or HKUST-1. (**B**) H&E staining of major organs after 7 days of treatment obtained from mice. Scale bar = 200 μm.

Biosafety and good biocompatibility are important considerations of artificial enzymes in potential biomedical and clinical applications. The toxicity assessment should be carried out first before clinical application. In this section, blood biochemistry analysis and histological analysis of major organs were further employed to assess the biosafety and biocompatibility of HKUST-1 in vivo. C57BL/6 male mice were chosen as animal models, and ten of them were randomly divided into two groups. PBS and HKUST-1 suspensions were injected into mice via the tail vein at a concentration of 0.5 mg mL^−1^ at a dose of 10 g/0.1 mL once a day for 5 days. Mice treated with PBS were used as parallel controls. After 7 days of different treatments, blood samples of the mice were collected for biochemistry analysis to study the toxicity of HKUST-1. As shown in Figure 5A, the biochemical indexes of the ratio of albumin to globulin (A/G), blood urea albumin (ALB), alanine transaminase (ALT), aspartate transaminase (AST), creatinine (CR), globulin (GLOB), total bilirubin (TBIL) and total protein (TP) were examined, demonstrating no significant difference between the PBS and HKUST-1 treatment groups. Finally, H&E staining of the major organs (heart, liver, spleen, lungs, and kidney) further confirmed the nontoxicity of HKUST-1 (Figure 5B). Taken together, HKUST-1 did not display obvious side effects on mice with biosafety and good biocompatibility, indicating its potential as an antibacterial and wound healing alternative.

## 3. Materials and Methods

### 3.1. Materials

Copper nitrate hydrate [Cu(NO_3_)_2_·xH_2_O], trimesic acid [C_6_H_3_(CO_2_H)_3_], and TMB were obtained from Aladdin Reagent Co., Ltd. (Shanghai, China). Polyvinylpyrrolidone [PVP, molecular weight (Mw) = 40,000] was obtained from Sigma–Aldrich Co., Ltd. Tetrakis (4-carboxyphenyl) porphyrin absolute (TCPP) was obtained from Tokyo Chemical Industry Co., Ltd. Sodium chloride, disodium hydrogen phosphate (Na_2_HPO_4_), potassium chloride (KCl), potassium dihydrogen phosphate (KH_2_PO_4_), and acetic acid (CH_3_COOH) were obtained from Sinopharm Chemical Reagent Co., Ltd. (Shanghai, China). Ethanol absolute and N,N-dimethylformamide (DMF) were purchased from Tianjin Damao Chemical Reagent Co., Ltd. H_2_O_2_ and methanol (CH_4_O) were purchased from Guangzhou Chemical Reagent Co., Ltd. (Guangzhou, China). Ultrapure water (18.2 MΩ; Millipore Co., United States) was used to prepare all solutions. All solutions were stored at room temperature at 25 ± 2 °C for further use. All reagents were of analytical grade and used without further purification.

### 3.2. Synthesis of Cu-MOFs

Cu-TCPP was obtained by the surfactant-assisted method [42]. First, 25 mg of copper nitrate (Cu(NO_3_)_2_·xH_2_O) and 100 mg of PVP were dispersed in a beaker containing a solution of DMF (45 mL) and absolute ethanol (15 mL) under stirring conditions to form a blue mixture. After that, 60 mg of TCPP was dissolved into the blue mixture under ultrasonication for 10 min. Then, the mixing solution was added to a Teflon autoclave and heated at 80 °C for 4 h. The resultant product was collected by centrifugation at 8000 rpm for 10 min. Finally, the red product was washed with absolute ethanol three times and dried at 60 °C.

The synthesis process of HKUST-1 was based on the previous literature [43]. Cu (NO_3_)_2_·xH_2_O (1.82 g) and C_6_H_3_(COOH)_3_ (0.875 g) were dissolved in a beaker containing 50 mL of absolute methanol with ultrasonication to obtain blue and transparent solutions, respectively. The copper nitrate solution was mixed with the trimesic acid solution under stirring. After that, the mixing solution was kept at 25 °C. After precipitating for 2 h, HKUST-1 was collected by centrifugation at 5000 rpm for 10 min. The obtained blue product was washed with methanol twice. Finally, the blue powder was dried under vacuum conditions for future use.

### 3.3. POD-like Catalytic Activity of Cu-MOFs

The POD-like activity of Cu-MOFs was systematically studied through the catalytic oxidation of TMB with the assistance of H_2_O_2_. The catalytic oxidation activity of Cu-MOFs was evaluated by gas bubble formation, the formation of a blue-colored oxTMB leading to color changes in the buffer solution and absorbance changes in oxTMB at 652 nm. In this experimental section, 20 mM acetate buffer (pH = 4.0) was used. Briefly, 1 mL HAc-NaAc buffer solution (20 mM, pH = 4.0) contained TMB, H_2_O_2_ and Cu-MOFs with final concentrations of 2 mM, 20 mM and 30 μg mL^−1^, respectively. The varying concentrations of Cu-MOFs, TMB, and H_2_O_2_ for the POD-like activities were explored. The POD-like activities of Cu-MOFs were assessed under the same conditions. Similarly, kinetics measurements were performed to analyze the catalytic activity of HKUST-1 by changing the concentrations of TMB and H_2_O_2_. The kinetic parameters were assessed by the Michaelis–Menten equation: $V_{0}$ = $V_{max}\times S/\left(K_{m}+S\right)$, where $V_{max}$ and $V_{0}$ correspond to the maximum velocity and initial rate, respectively. S and $K_{m}$ represent the concentration of the substance and the Michaelis–Menten constant, respectively.

### 3.4. Detection of •OH

The generation of •OH, catalyzed by Cu-MOFs, was verified by fluorescence (FL) experiments. The hydroxyl radical assay was as follows: the solutions in the tubes including (I) TA, (II) TA + H_2_O_2_, (III) Cu-TCPP, (IV) HKUST-1, (V) TA + H_2_O_2_ + Cu-TCPP and (VI) TA + H_2_O_2_ + HKUST-1 were reacted for 12 h in the dark. After that, the supernatant was collected by centrifugation at 8000 rpm for 10 min. Then, the fluorescence spectra at 435 nm of the supernatant were recorded. The final concentrations of TA, H_2_O_2_ and Cu-MOFs were 0.5 mM, 10 mM and 30 μg mL^−1^, respectively.

### 3.5. Bacterial Culture and Antibacterial Assay In Vitro

*E. coli* (ATCC 25922) and MRSA (ATCC 43300) bacterial cells in the log phase were used in the following experiments. The bacterial conditions were detected by measuring the OD600 nm using a UV–Vis spectrophotometer. The value of OD600 nm is approximately 0.5. Bacterial cells in the log phase can be used in the following experiments. The bacterial suspension was diluted with 0.01 M sterilized PBS to 1 × 10^7^ CFU mL^−1^ for future use.

For the antibacterial experiments, the plate counting method was chosen to determine the sterilization effect of Cu-MOFs. The treatments were divided into six groups: (I) PBS, (II) H_2_O_2_, (III) Cu-TCPP, (IV) Cu-TCPP + H_2_O_2_, (V) HKUST-1, and (VI) HKUST-1 + H_2_O_2_. The mixtures of all groups were incubated for 3 h at 37 °C. Then, 100 μL mixtures of all groups were spread on LB solid medium and incubated at 37 °C for 12 h. The number of colonies was counted. All experiments were repeated three times.

Fluorescence-based live/dead bacteria staining analysis was employed to investigate the antibacterial performance of HKUST-1 with the assistance of H_2_O_2_. *E. coli* and MRSA, treated with PBS or HKUST-1+ H_2_O_2_, were stained with SYTO and PI for 30 min in the dark. Afterward, the bacteria were washed and collected by centrifugation at 12,000 rpm three times. Finally, fluorescence microscopy was employed to observe the stained bacterial cells.

The morphological changes in the bacteria were characterized by scanning electron microscopy to determine the sterilization effect of PBS or HKUST-1 + H_2_O_2_ treatment. First, bacteria treated with HKUST-1 + H_2_O_2_ were used for the treated group, and PBS treatment was used for the blank control. After incubation at 37 °C for 3 h, the bacteria were collected by washing and centrifugation and fixed with 4% paraformaldehyde. The bacteria were washed thoroughly with DI water, followed by dehydration using a series of ethanol solutions. Finally, morphological changes in treated bacteria were examined under conditions of SEM after sputter-coating with gold.

### 3.6. Evaluation of Antibacterial Activity In Vivo

Animal experiments were designed based on the standard protocol and approved by the Ethical Review Committee and Laboratory Animal Welfare Committee of Guangdong Pharmaceutical University (gdpulacspf2017523). A full-thickness cutaneous wound with MRSA infection model was built in male C57/BL6 mice (8–10 weeks, 25–30 g, six-ten mice per group) purchased from Guangdong Laboratory Animal Center. A round wound, with a diameter of approximately 8 mm, was created on the back of each mouse. Twenty mice were divided into four treatment groups: (I) PBS + 3% CMC-Na hydrogels, (II) H_2_O_2_ + 3% CMC-Na hydrogels, (III) HKUST-1 + 3% CMC-Na hydrogels and (IV) HKUST-1 + H_2_O_2_ + 3% CMC-Na hydrogels. The final concentrations of H_2_O_2_ and HKUST-1 were 10^−3^ M and 2 mg mL^−1^, respectively. After sterilization with 75% alcohol and shaving with hair removal cream, 50 μL 1 × 10^7^ CFU mL^−1^ of MRSA suspension was dropped on the wound site to construct the infectious wound model of the mouse. The wounds were observed and photographed by a digital camera. The hydrogels were changed at daily intervals after physiological saline solution irrigation. After 7 days of treatment, the mice were sacrificed. The related wound tissues were harvested for histological evaluation. The wound closure rate was calculated by the following equation:

Wound closure rate = Wound Area _(Day0–DayX)_/Wound Area _(Day0)_ × 100%

### 3.7. Biosafety Assay In Vivo

Ten mice were divided into two groups: (I) HKUST-1 treatment was used for the treated group, and (II) PBS solution treatment was used for the blank control group. After 7 days of tail vein injection of HKUST-1 suspension (0.5 mg/mL) at a dose of 10 g/0.1 mL, two groups of mice were sacrificed. The main organ tissues were harvested for histological evaluation, and blood samples were collected for biochemical analysis.

## 4. Conclusions

In summary, we reported Cu-MOFs in different dimensions for antibacterial application and wound healing promotion. Cu-TCPP with a sheet-like and layer-by-layer ultrathin 2D structure and HKUST-1 with a uniform 3D structure in an octahedral shape were synthesized successfully. Benefiting from the uniform 3D structure, HKUST-1 exhibited higher POD-like activity than Cu-TCPP. Based on the excellent POD-like activity, HKUST-1 could greatly accelerate the generation of ROS in terms of the decomposition of H_2_O_2_ into highly toxic •OH. The POD-like activity studies and fluorescent experiments systematically confirmed the results. Inspired by POD-like activity and the generation of •OH, Cu-MOFs were employed to investigate the antibacterial performance and wound healing in vivo. HKUST-1 can catalyze H_2_O_2_ toward •OH generation for *E. coli* and MRSA disinfection in vitro, suggesting broad antimicrobial spectrum effects that could be ascribed to a large amount of •OH generation disrupting the bacterial antioxidant system. Moreover, HKUST-1 could accelerate wound healing without significant biological toxicity to major organs or side effects. Therefore, this work has provided MOFs with different structural dimensions, having variable POD-like activity as a new antimicrobial strategy, and expanded the potential applications of MOFs in biomedical and clinical fields.

## Figures and Tables

**Table 1:** Comparison of the Michaelis–Menten ($K_{m}$) constant and maximum reaction rate ($V_{max}$) of HKUST-1.

Catalyst	Substance	$K_{m}\;(\mathrm{mmol}\;L^{-1})$	Vmax (mol L^−1^ s^−1^)	References
ZIF-67	TMB	13.69	3.5 × 10^−7^	[35]
	H_2_O_2_	3.52	2.8 × 10^−7^	
Antibody@Cu-MOFs	TMB	3.91	5.445 × 10^−7^	[36]
	H_2_O_2_	7.37	1.075 × 10^−7^	
Cu-MOFs (CuCl_2_)	TMB	4.11	5.556 × 10^−7^	[36]
	H_2_O_2_	6.41	1.02 × 10^−7^	
Cu-MOFs (Cu(NO_3_)_2_)	TMB	2.56	2.5 × 10^−7^	[37]
	H_2_O_2_	4.34	1.82 × 10^−7^	
Cu-MOFs (Cu(NO_3_)_2_)	TMB	0.456	2.478 × 10^−8^	[38]
	H_2_O_2_	28.58	5.45 × 10^−8^	
Cu-MOFs (CuI)	TMB	2.4862	7.517 × 10^−8^	[39]
	H_2_O_2_	0.163	6.736 × 10^−8^	
CuFe_2_O_4_	TMB	2.26	2.07 × 10^−8^	[9]
	H_2_O_2_	0.5	2.61 × 10^−8^	
(Ni_2_Co_1_)_0.5_Cu_0.5_ MOFs	TMB	0.34	1.81 × 10^−8^	[10]
	H_2_O_2_	1.08	1.29 × 10^−8^	
AuNPs/Cu-MOFs	TMB	0.29	2.96 × 10^−7^	[40]
	H_2_O_2_	0.65	2.25 × 10^−7^	
HRP	TMB	0.434	10.0 × 10^−8^	[41]
	H_2_O_2_	3.702	8.71 × 10^−8^	
HKUST-1	TMB	0.545	0.833 × 10^−8^	This work
	H_2_O_2_	2.036	1.757 × 10^−8^	

## Data Availability

The data used to support the findings of this study are available from the corresponding author upon request.

## Supplementary Materials

The following supporting information can be downloaded at: https://www.mdpi.com/article/10.3390/ijms24043173/s1.
