# Supplementary material for: Different Dimensional Copper-Based Metal–Organic Frameworks with Enzyme-Mimetic Activity for Antibacterial Therapy
Source: Int J Mol Sci. 2023 Feb 6;24(4):3173. doi: 10.3390/ijms24043173 (PMC9967080; doi:10.3390/ijms24043173)
Supplement: Supplementary file 1 [file ijms-24-03173-s001.zip › ijms-2127827-supplementary.pdf]

# Supplementary Materials

## Different Dimensional Copper-Based Metal-Organic Frameworks with Enzyme-Mimetic Activity for Antibacterial Therapy

Chuyan Lin [a,b,#], Xiangjian Guo [a,c,#], Fayin Mo [a], Duanping Sun [a,b,*]

[a] Guangdong Provincial Key Laboratory of Pharmaceutical Bioactive Substances, Center for Drug Research and Development, Guangdong Pharmaceutical University, Guangzhou 510006, Guangdong, China

[b] Key Specialty of Clinical Pharmacy, The First Affiliated Hospital of Guangdong Pharmaceutical University, Guangzhou 510699, Guangdong, China

[c] The First Affiliated Hospital of Sun Yat-Sen University, Guangzhou 510080, Guangdong, China

[#] These authors contributed equally to this work.

* Corresponding author.

E-mail addresses: sundp@gdpu.edu.cn; sunduanping@126.com (D. Sun)

## S1.1. Apparatus and Instrumentation

The different morphologies and structures of HKUST-1 were characterized by transmission electron microscopy (TEM 1400, Japan) and scanning electron microscopy (SEM, Regulus 8230, Hitachi, Japan). An accelerating voltage of 3 kV and a magnification of 10×-20× were used. The corresponding infrared spectra were obtained using Fourier transform infrared (FT-IR) spectra obtained on a Fourier transformation infrared spectrometer (IR, EQUINOX 55, Germany). X-ray powder diffraction (XRD) patterns were recorded on a PANalytical instrument (Empyrean, Netherlands) to examine the crystal phase of the samples. The surface composition and valence states were studied by X-ray photoelectron spectroscopy (XPS, Nexsa, Thermo Fisher Scientific, United States). The UV–Vis absorption spectra were recorded by a UV–Visible spectrophotometer (Shimadzu UV-2450, Japan). Fluorescence measurements were carried out by a spectrofluorometer (Shimadzu RF-5301PC, Japan).

## S1.2. Supplementary Figures

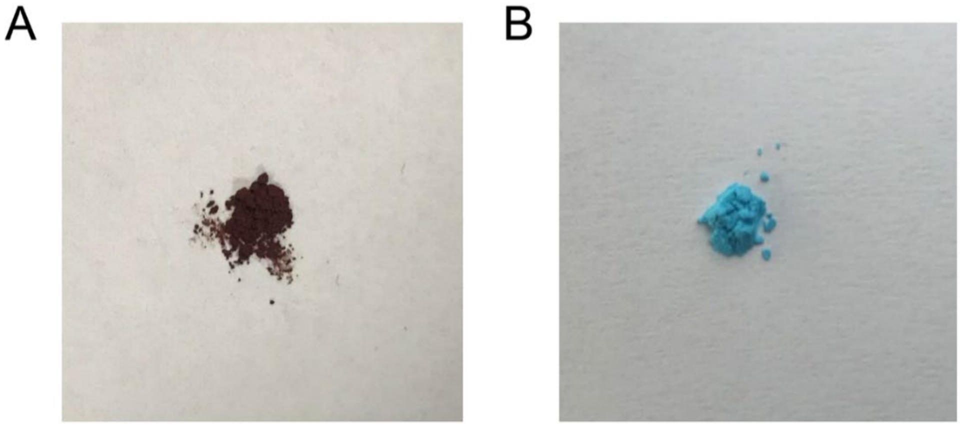


**Figure S1.** Photographs of (A) Cu-TCPP and (B) HKUST-1 powders.

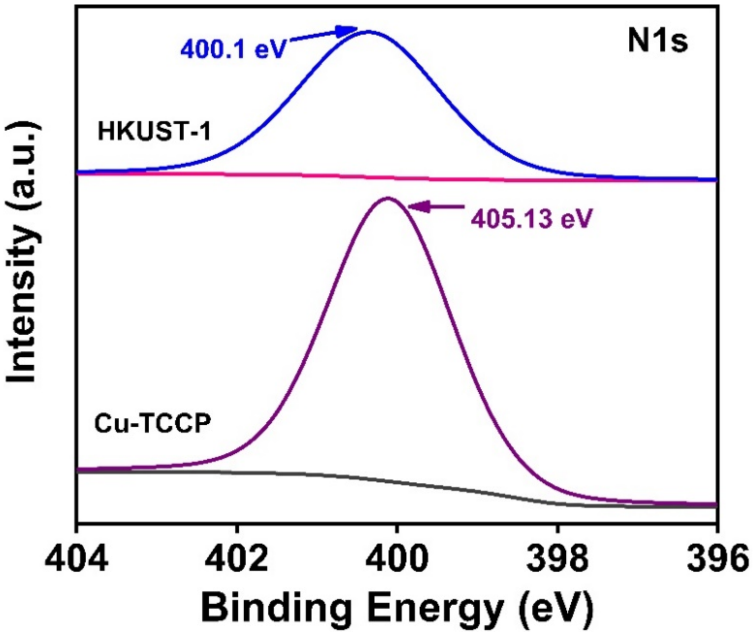


**Figure S2.** High-resolution XPS spectra of N 1s for HKUST-1 and Cu-TCPP.

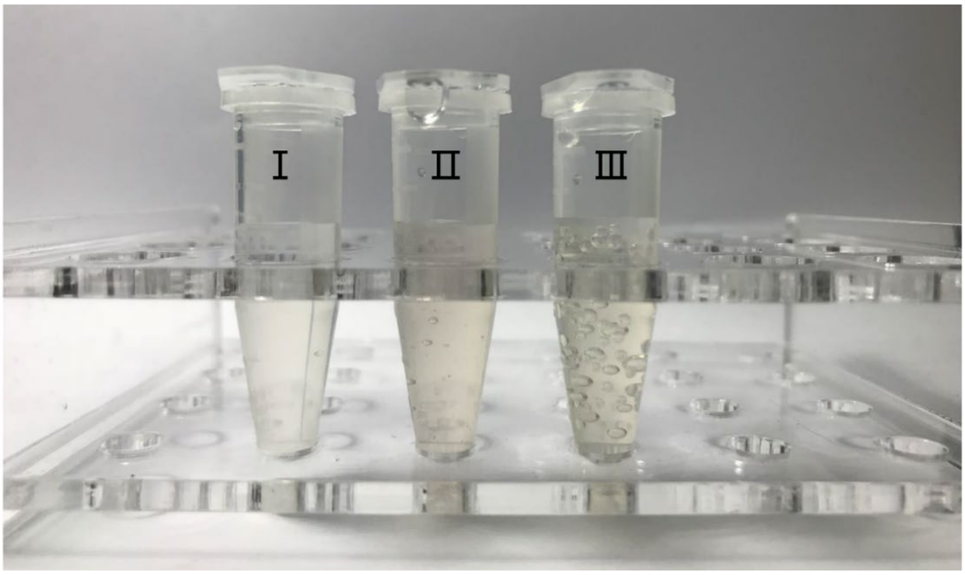


**Figure S3.** A photo of tubes containing different solutions in 0.02 M HAC-NaAC solution containing 1 M $H_2O_2$ after reacting with (I) blank, (II) Cu-TCPP and (III) HKUST- for 15 min.

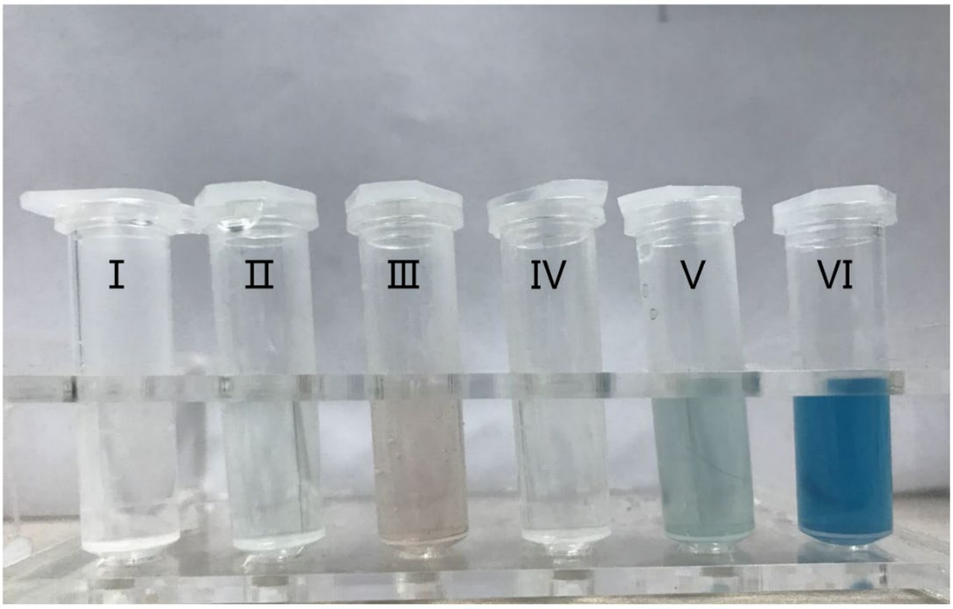


**Figure S4.** Visual color changes of TMB in different reaction systems after reacting for 15 min: (I) TMB, (II) TMB+$H_2O_2$, (III) Cu-TCPP, (IV) HKUST-1, (V) TMB+ $H_2O_2$+Cu-TCPP and (VI) TMB+ $H_2O_2$+HKUST-1.

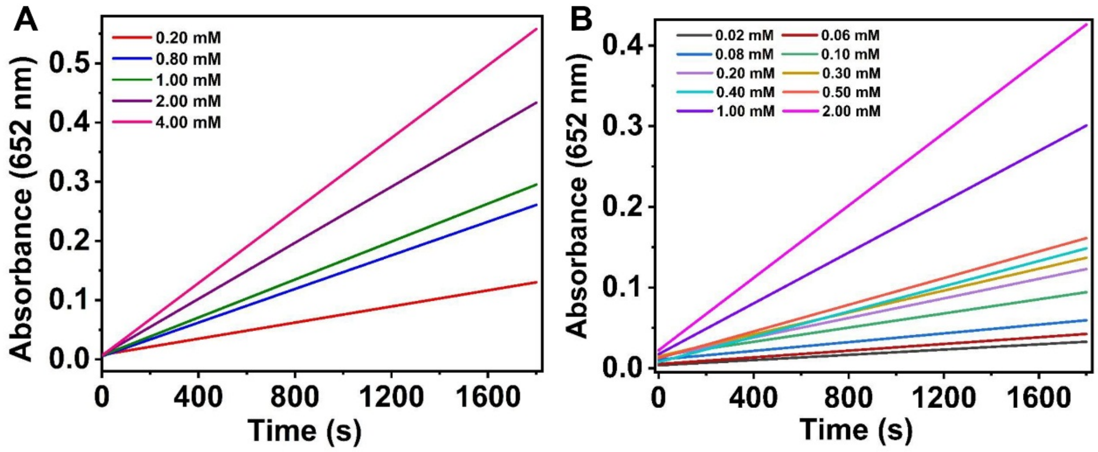


**Figure S5.** Kinetic curves of (A) $H_2O_2$ and (B) TMB at various concentrations. The absorbance at 652 nm was used.

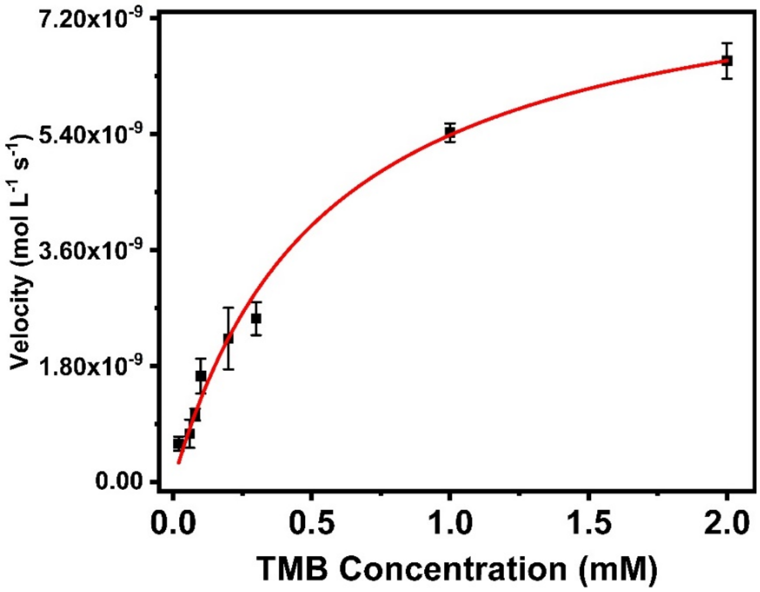


**Figure S6.** The steady-state kinetic assays of HKUST-1 for TMB.

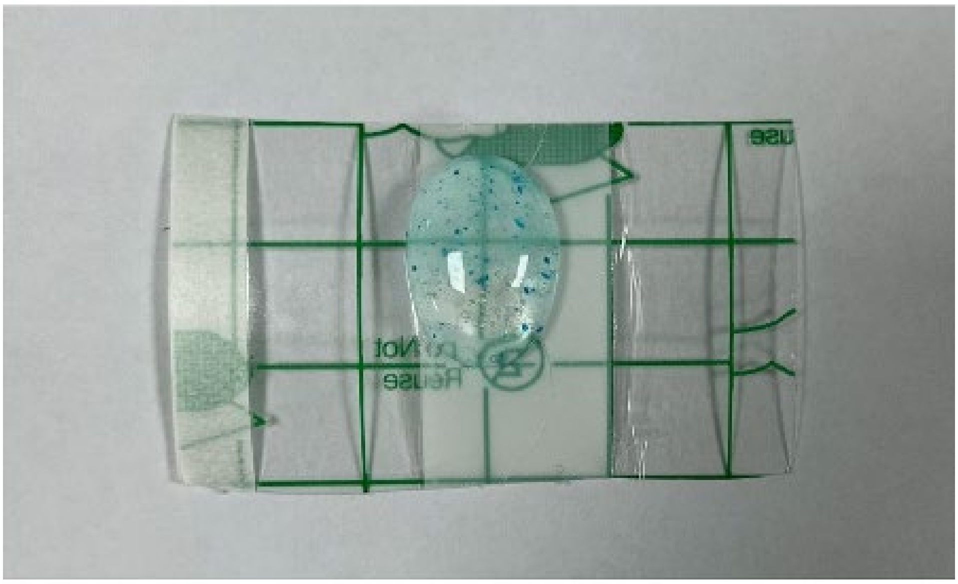

**Figure S7.** Photograph of dressing containing HKUST-1 materials.
